# *Nocardia* Brain Abscess and CD4^+^ Lymphocytopenia in a Previously Healthy Individual

**DOI:** 10.1155/2015/374956

**Published:** 2015-09-16

**Authors:** Norair Adjamian, Adeline Kikam, Kathryn Ruda Wessell, Jason Casselman, Erin Toller-Artis, Olapeju Olasokan, Robert W. Hostoffer

**Affiliations:** ^1^Kansas City University of Medicine & Biosciences, Kansas City, MO 1750, USA; ^2^Firelands Regional Medical Center, Sandusky, OH 1111, USA; ^3^University Hospitals Richmond Medical Center, Richmond Heights, OH 27100, USA

## Abstract

*Nocardia* brain abscesses are a known occurrence in patients with immunocompromised conditions. Nocardial infection is commonly an unfortunate sequela to other complications which these patients are being followed up and treated for. The incidence of nocardial brain abscess in an otherwise healthy patient is extremely rare. We present a case of *Nocardia* brain abscess in a previously healthy individual, who, upon workup for vision and gait abnormalities, was shown to have multiple brain abscesses and a decreased absolute CD4^+^ lymphocyte count. Adding to the rarity of our case, the finding of lymphocytopenia in our patient was unrelated to any known predisposing condition or infectious state.

## 1. Introduction

Patients with deficiencies in cell-mediated immunity are at high risk of infection with opportunistic pathogens such as* Nocardia* [1]. Among these individuals are those with lymphoma, various malignancies, and human immunodeficiency virus (HIV), organ or hematopoietic stem-cell transplant recipients, and those receiving long term treatment of medications that suppress the immune system [1]. Of other known described conditions, Idiopathic CD4^+^ Lymphocytopenia (ICL) was first defined in 1992 by the US Centers for Disease Control and Prevention (CDC) as

… a documented absolute CD4 T-lymphocyte count of <300 cells/mm3 or <20% of total T-cells on two separate time points at least six weeks apart, without evidence of infection of HIV-1 or HIV-2 testing, and without immunodeficiency or therapy related to decrease of CD4 T-cells… [2].

We report the case of a patient with depressed CD4^+^ counts, unrelated to HIV infection or other immunocompromising conditions, presenting with* Nocardia* brain abscess. Her CD4 T-lymphocyte count, although not to the strict definition of <300 cells/mm^3^, was clearly depressed from the normal value range and idiopathic in nature.

## 2. Case Presentation

At the age of 70, our patient presented to the Emergency Department (ED) with slurred speech, left peripheral visual field deficits, left sided weakness, and unsteady gate. Her past medical history includes asthma, allergic rhinitis, arthritis, and hypotension. Computed Tomography (CT) of the brain revealed large, low-density areas in the right temporal, occipital, and parietal regions, primarily involving the white matter. Magnetic Resonance Imaging (MRI) showed multiple ring enhancing lesions within the right hemisphere, with the dominant lesion found in the posterior parietal-occipital region, measuring 3.2 × 2.4 cm. Chest X-ray revealed mild left lower lobe density indicative of subsegmental atelectasis, but otherwise the lungs appeared clear. She underwent a right craniotomy with excision of the right occipital mass that confirmed abscess formation, culture positive for* Nocardia farcinica*. The patient was placed on intravenous (IV) antibiotics consisting of trimethoprim/sulfamethoxazole (TMP/SMX) and linezolid for two months following her diagnosis and then transitioned to oral TMP/SMX. She remains on TMP/SMX for prophylactic therapy. HIV-1 and HIV-2 antibodies were nonreactive 2 months after surgery. CD4^+^ counts acquired 6 months and 11 months after surgery revealed depletion of CD4^+^ T cells with a declining pattern. The values were 398 cells/uL and 343 cells/uL, respectively. The remainder of her immunodeficiency workup was negative, including no demonstrable Cluster Differentiation (CD) markers that revealed an additional underlying immunodeficiency. Malignancy workup was also negative.

## 3. Discussion

*Nocardia* is described as an opportunistic bacteria, most commonly infecting immunocompromised patients but not uncommon in immunocompetent individuals as well [1]. Those at particularly high risk are patients with deficiencies in cell-mediated immunity [1]. The most common anatomic site for nocardial infection is pulmonary due to inhalation being the primary route of exposure [1]. Classic pulmonary symptoms include cough, shortness of breath, chest pain, hemoptysis, fever, night sweats, and fatigue. Pulmonary nocardiosis is associated with patients with underlying pulmonary diseases including asthma, chronic sarcoidosis, emphysema, and chronic bronchitis, who have been treated with long term, high dose of corticosteroid therapy [3]. Observed sites for extrapulmonary nocardiosis include the central nervous system and skin [3]. CNS manifestations include multiple brain abscesses, which can present with headaches, nausea, vomiting, seizures, or other neurological symptoms [3]. Cutaneous nocardiosis may lead to superficial abscesses or localized cellulitis [3].

Similar to our reported case, patients with Idiopathic CD4^+^ Lymphocytopenia (ICL) go undiagnosed until they develop symptoms suggestive of opportunistic infections [4]. Ahmad et al. found 258 diagnosed cases of ICL in 143 published papers [4]. This study was able to determine the ten most common opportunistic organisms to infect these patients:* Clostridium neoformans* (26.6%),* Mycobacterium* spp. (17%),* Candida* spp. (16.20%), Varicella Zoster Virus (13.10%), Human Papilloma Virus (11.60%), Herpes Simplex Virus (8.10%),* Pneumocystis carinii* pneumonia (7.70%), Cytomegalovirus (5.80%), John Cunningham Virus (3.90%), and* Toxoplasma* (3.10%) [4]. Among the unusual bacterial infections noted, there were only two nocardiosis cases. One, similar to our patient, was associated with* N. farcinica* brain abscesses, and the second nocardiosis case was disseminated* N. asteroides* without brain abscesses [5]. The patient with the* Nocardia* brain abscesses was a 19-year-old heterosexual male who had accompanying epidermodysplasia verruciformis-like skin eruptions with dysplasia by HPV-16, along with pulmonary tuberculosis [5]. The patient died of respiratory failure during treatment [5]. After a thorough review of the literature, the case of* N. farcinica* brain abscess of the 19-year-old male was the only case of* Nocardia* brain abscesses reported in a patient with Idiopathic CD4^+^ Lymphocytopenia [5].

We present, to our knowledge, the second reported case of* Nocardia* brain abscess in a patient with depressed absolute CD4^+^ counts, unrelated to HIV or other conditions depleting cell mediated immunity. In contrast to the first ICL* Nocardia* brain abscess case, our patient had no underlying pulmonary comorbid disease besides her mild, well-controlled asthma. The 19-year-old male with the first reported* Nocardia* brain abscess case with ICL had underlying pulmonary tuberculosis. Our patient's CD4 T-lymphocyte count, although not to the strict definition of <300 cells/mm^3^, was clearly depressed (down to 343 cells/uL) from the normal value range. Prior to her presentation at the Emergency Department, our patient had no major illnesses, and her comorbid conditions of asthma, allergic rhinitis, arthritis, and hypotension were all easily managed, and without complications. Similar to other reported cases of CD4^+^ T-lymphopenia, her indolent condition went unrecognized until the* Nocardia* brain abscess manifested as acute neurologic symptoms.

## 4. Conclusion

*Nocardia* is a rare opportunistic pathogen found in immunocompromised patients. Nocardial brain abscesses in patients with idiopathic CD4^+^ lymphopenia are exceedingly rare in the literature. To our knowledge, we present the first case of a nocardial brain abscess in an otherwise healthy patient with depressed CD4^+^ counts idiopathic in nature. She did not present with other coinfections, displayed symptomatology limited to neurologic manifestations, demonstrated infection clearance with abscess resection and antibiotic therapy, and has remained infection-free since TMP/SMX prophylaxis was initiated.
